# Novel PEEK Retentive Elements versus Conventional Retentive Elements in Mandibular Overdentures: A Randomized Controlled Trial

**DOI:** 10.1155/2022/6947756

**Published:** 2022-02-28

**Authors:** M. Y. Sharaf, Asharaf Eskander, Mohamed Afify

**Affiliations:** ^1^Department of Prosthodontics, Faculty of Dentistry, University of Menoufia, Shibin Al Kawm, Egypt; ^2^Department of Prosthodontics, Faculty of Dentistry, University of Cairo, Cairo, Egypt

## Abstract

**Background:**

Many patients suffer from lack of retention of conventional mandibular overdentures due to loss of clip retention over time. Computer-aided design-computer-aided manufacturing (CAD-CAM) milled polyether ether ketone (PEEK) materials may be used for the construction of retentive housing and clips for improving retention of implant-supported overdentures.

**Objective:**

To compare retention and patient satisfaction of implant-supported mandibular overdentures retained by conventional nylon clip and metal housings for ball attachments versus PEEK clip and housings.

**Methods:**

Twenty-two participants were divided into 2 equal groups (*n* = 11). The conventional group received implant-supported mandibular overdentures retained by metal housings and nylon retentive elements, while the PEEK group received implant-supported mandibular overdentures retained by PEEK retentive elements and housings. The PEEK retentive elements were made using computer-aided design and computer-aided manufacturing (CAD-CAM). The evaluation included measuring the retention by applying a gradual pulling up force by force meter and patient satisfaction with a 7-point visual analog scale (VAS) at overdenture insertion and 3, 6, and 12 months subsequently by a research interviewer.

**Results:**

The PEEK group showed statistically significantly increased retention force (*P* < 0.05) at the time of insertion (37.6/17.79) and after 3 months (33.9/16.78), 6 months (32.7/15.97), and 12 months (31.65/13.05). The conventional group had a statistically significantly higher mean overall satisfaction (*P* < 0.05) at the time of insertion (65/82.18). No statistically significant difference was found after 3 months (87.81/84.72). The PEEK group showed statistically significantly higher mean overall satisfaction (*P* < 0.05) after 6 months (86.36/80.18) and 12 months (85.45/79.54).

**Conclusions:**

According to the results of this study, the PEEK retentive material provided more retention than did the conventional material and led to improved patient satisfaction. The study was registered at clinical trials.gov (https://clinicaltrials.gov/ct2/show/NCT05079048).

## 1. Introduction

Implant-retained overdentures offer a reliable treatment to improve denture retention and patient quality of life, as it is considered as the standard treatment for the edentulous mandible [1–3]. Approximately 60% and up to 85% of implants placed in completely edentulous patients are for the prostheses with different types of attachments and have been successfully used in edentulous patients [4–6]. The attachments between the implants and the prosthesis typically require frequent adjustments and repairs, with the attachment components liable to fracture, distortion, and disengagement with gradual loss of retention and stability. These problems are a common cause of patient complaints [7–10]. Ball and socket attachments were used in many overdentures. Ball attachment requires smaller space within prostheses, easy cleaning, more economical and less sensitive technique, and it distributes and reduces the transmitted load to the implant by allowing slight multidirectional movement [11–13].

Polyether ether ketone (PEEK) represents a modification of the thermoplastic high-performance polymer group polyether aryl ketone (PEAK). PEEK is a high-temperature thermoplastic polymer with a melting point of about 343°C, a density of 1.3 to 1.5 g/cm^3^, and an elastic modulus between 3 GPa and 4 GPa compared with titanium of 113 GPa and zirconia of 204 GPa [14–17]. In addition to high thermal stability and high hardness, PEEK has low water absorption and solubility. Therefore, PEEK is an interesting alternative to traditional alloy and ceramic dental materials. Biofilm formation on PEEK surfaces is similar to or lower than that on other prosthodontic materials such as titanium and zirconia, and its low surface energy provides resistance to surface modifications by chemical treatment [18–20]. PEEK has been used for dental implants, interim abutments, framework material for removable dental prostheses, and fixed partial dentures and crowns [21–24]. PEEK can be processed by CAD-CAM milling from blanks or by vacuum pressing [25].

The purpose of this clinical trial was to compare the retention and patient satisfaction of implant-supported mandibular overdentures retained using conventional nylon clip and metal housings for ball attachments versus PEEK clip and housings. The research hypothesis was that the PEEK retentive elements would provide better retentive force as well as better patient satisfaction.

## 2. Materials and Methods

Twenty-two participants were enrolled according to the following criteria: edentulous class I or II PDI classification, angle class I maxillo-mandibular relationship using facial profile, patients complaining of reduced denture retention, and wearing dentures for more than one year. All patients were free from neuromuscular disorders and temporomandibular joint disorders. In contrast, the exclusion criteria were patients who smoke more than 10 cigarettes per day and patients with any systemic disease that directly affects bone metabolism and healing, such as uncontrolled diabetes mellitus. The study was approved by the ethical committee and adhered to the principles of the Declaration of Helsinki. The study was registered at Clinical trials.gov (https://clinicaltrials.gov/ct2/show/NCT05079048).

### 2.1. Sample Size

Sample size calculation was carried out using the comparison of retention force values between the new and the ball attachment. As reported in the previous publication [26], the mean ± SD of retention force after 12 months in the ball group was approximately 44.25 ± 3.3, and we assumed that the minimal clinically important difference is 10% improvement by using the new retentive element. Accordingly, we calculated that the minimum proper sample size was 11 participants in each group to achieve 80% power at *α* = 0.05 level using Student's *t*-test for independent samples. Sample size calculation was carried out using StatsDirect statistical software version 2.7.2 for MS Windows, StatsDirect Ltd., Cheshire, UK.

The participants were randomly (sealed envelope technique) divided into 2 equal parallel groups (*n* = 11). The allocation concealment key was retained by the chairman of the department. The conventional group received implant-supported mandibular overdentures retained using metal housings and nylon retentive clip for the ball attachments (Superline; Dentium.Korea). The PEEK group received implant-supported mandibular overdentures retained using PEEK retentive elements and housings for ball attachments.

Preliminary maxillary and mandibular impressions were made with alginate impression material (Hydrogum 5; Zhermack SpA) and poured to obtain diagnostic casts. Maxillary and mandibular custom acrylic resin trays (Acrostone Dental & Medical Supplies) were fabricated on the diagnostic casts, border molding was carried out, and definitive impressions were made with zinc oxide eugenol impression material (Cavex Holland). Occlusal rims and record bases were fabricated. A maxillary face-bow record was made and transferred to a semiadjustable articulator (Standard Face Bow, and A7 Articulator; Bio Art Brazilian) to mount the maxillary casts. Centric jaw relation records were recorded to mount mandibular casts using the wax wafer technique [27]. Acrylic resin semianatomical teeth (Acrostone Dental & Medical Supplies) of appropriate shape, size, and shade were selected and arranged according to the lingualized occlusal concept [28], and waxing the trial dentures was completed. Try-in of waxed dentures was carried out intraorally. The dentures were cured, finished, and polished. At the insertion visit, the dentures were evaluated intraorally for extension, retention, stability, esthetics, phonetics, occlusal plane orientation, centric occluding relation, and vertical dimension as each step was carried out by the same operator to ensure standardization of all dentures. Then the participants were instructed on denture and oral hygiene measures [daily mouthwashes (Macro Group Pharmaceutical), dentures should be cleaned daily by soaking and brushing with a nonabrasive denture cleanser (Corega GlaxoSmithKline group)].

The mandibular dentures were duplicated for use as radiographic stents using a mixture of acrylic resin and barium sulfate in a 4 : 1 ratio. The stents were evaluated intraorally for adaptation. Cone-beam computed tomography (CBCT) images were made with the radiographic stents in place to determine the optimal sites (in the canine area) for implant placement. The radiographic stents were converted into surgical guides by making holes in the proposed implant sites [29, 30].

**Figure 1:**
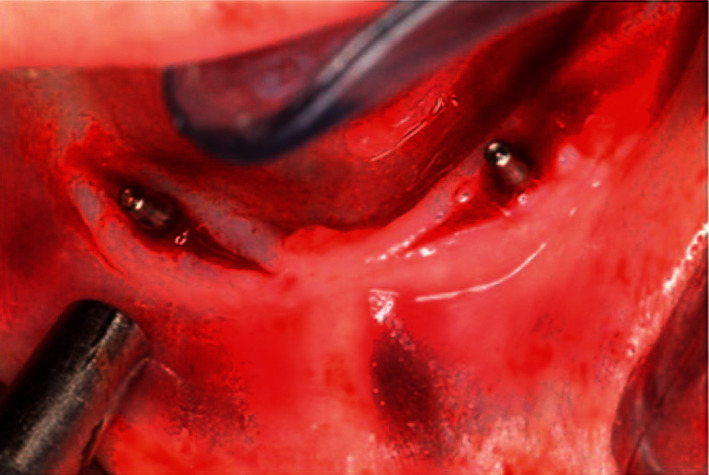
Checking parallelism between implants by the parallel pins.

Local anesthesia (Inibsa Artinibsa 4%.Spain) was given, and the surgical guides were placed, and an explorer was used to mark the proposed sites for implant placement. A crestal incision was made using a Bard-Parker blade no.15, extending 5 mm mesial and distal to the marked implant sites. A full-thickness mucoperiosteal flap was reflected using a sharp mucoperiosteal elevator. In some situations, with a sharp knife-edge ridge or irregular ridge, a low-speed fissure bur and bone file were used for smoothing the ridge and creating a bony plateau. The surgical guides were again placed in the patient's mouth, and a large round bur (carbide bur; Mani Japan) was used to mark the site of implant placement in the bone under copious saline irrigation. The implant osteotomies were drilled using an electric motor and a reduction handpiece (1 : 20) (Implant X cube; Saeshin. Korea). Pilot drills were used to drill osteotomies 10 mm in depth, followed by sequential drills of larger diameters until the final implant diameter (3.6 mm) was reached. The same procedure was repeated for the second implant, and then parallelism was evaluated among both implants using a parallel tool (Superline; Dentium) (Figure 1). After the preparation of the osteotomies, the implants (Superline; Dentium) were placed into the osteotomies and rotated gradually using the ratchet (Superline; Dentium) with torque of 35 Ncm in most cases. After the implants were completely seated, the implant mount was removed; the hexagon driver was used to place the cover screw in its place. The flap was irrigated with sterile saline solution, repositioned, and sutured with interrupted sutures using 0000 black silk suture material (Egysorb; surgical suture).

**Figure 2:**
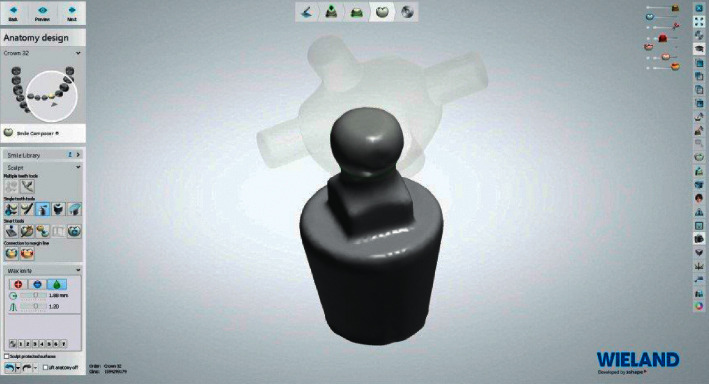
Scanned image of ball abutment analog.

**Figure 3:**
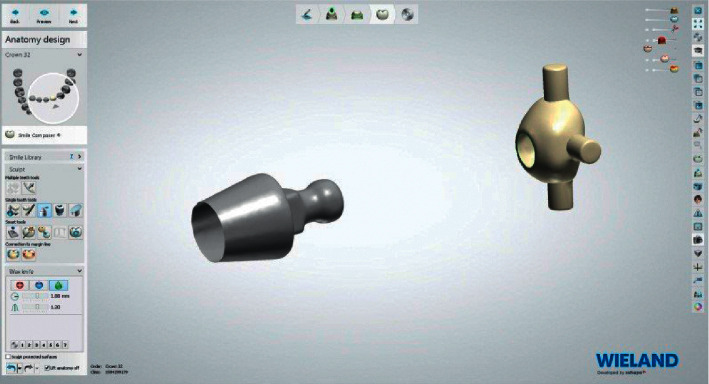
PEEK cap and ball abutment design.

**Figure 4:**
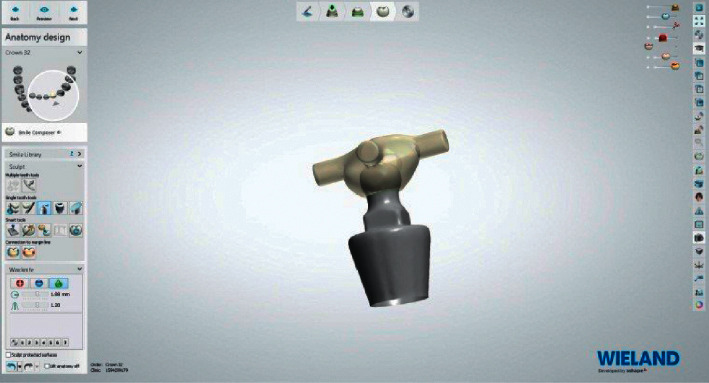
PEEK cap adapted to ball abutment.

The process of fabrication of PEEK retentive elements and housing started by scanning the ball abutment (Superline; Dentium) with a laboratory dental scanner (Lab scanner; 3shape) to obtain an image of the ball abutments (Figure 2). The design was carried out on dental software (Dental System 2016; 3Shape) to produce the PEEK retentive elements and housings, that were adapted to ball abutments (Figures 3 and 4).

After designing the PEEK retentive element and housings, they were evaluated by different features of the software to make sure that all parameters were carried out as predetermined in the designing phase to ensure an intimate fit between the PEEK retentive element and housing from one side and the ball abutment. This standard tessellation language (STL) file was transferred to the CAM software (Zenotec CAM; Wieland Dental) and to be milled (PEEK discs (DD peek MED: Dental Direkt GmbH) with a dimension of 98.5 × 14 mm) on the 5axis milling machine (Zenotec Select Hybrid; Wieland Dental). It took around 10 minutes to mill a single retentive element; then, they were cut from their sprue-like attachments from the disc, and the excess flashes were removed.

After 3 months, the participants were recalled, and the following procedures were carried out: Digital periapical radiography was performed to assess the supporting bone around implants. Proper cleaning of the field was carried out using chlorhexidine mouthwash. Field block anesthesia was given around the implant sites. Healing abutments (Superline, Dentium. Korea) were inserted and screwed into the implants using a hex driver. The healing abutments were left for 10 days for gingival healing and the formation of a gingival collar and then replaced by the ball abutment of the suitable transmucosal height from (1 to 3 mm).

**Figure 5:**
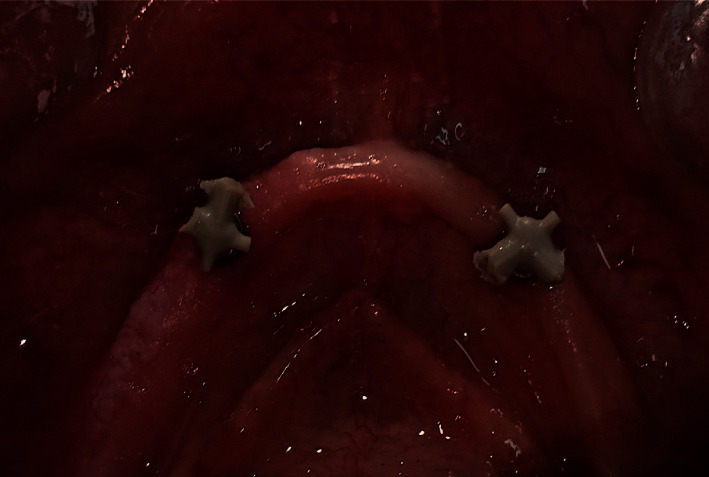
PEEK inserts attached to the ball abutment.

**Figure 6:**
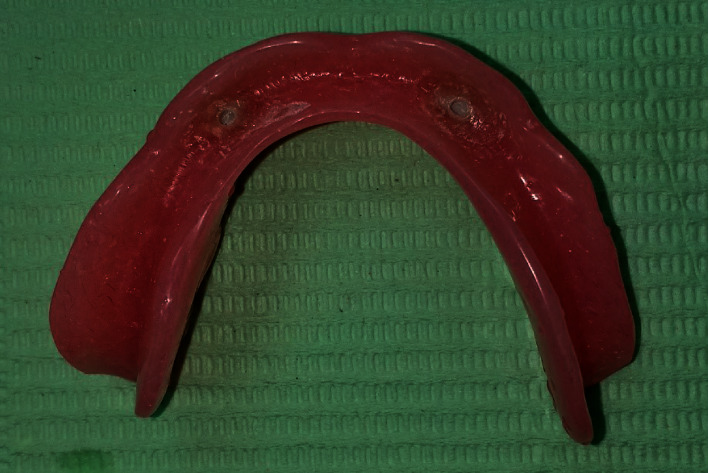
Fitting surface with picked up PEEK housing.

**Figure 7:**
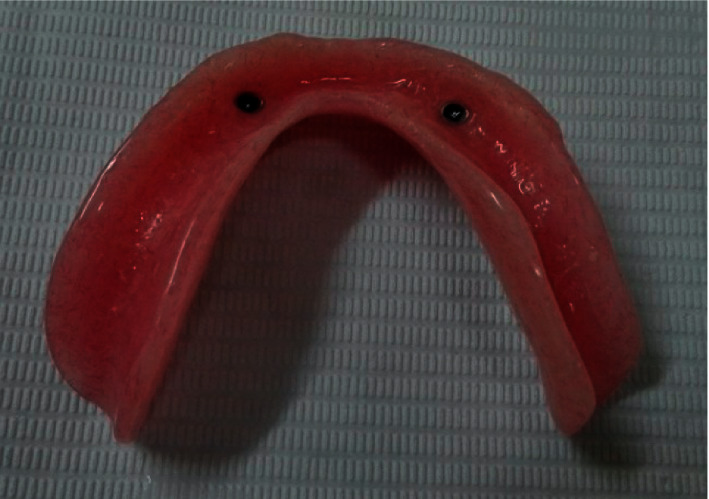
Fitting surface with picked up metal housing.

Pick up of PEEK retentive elements (Figures 5 and 6) and the metal housings, retentive caps (Figure 7), were carried out in the denture base using self-curing acrylic resin.

Retention was measured at the time of insertion and 3, 6, and 12 months after prosthesis insertion for all participants. The measurement was taken using the identification of the geometric center of the mandibular arch to allow placement of the metallic loop. The force meter machine (Eagle: ELT 3000) was attached to the metallic loop to measure the retentive force of mandibular overdentures. Gradual pulling up force was applied to the metallic loop of the denture until it disengaged. The record that appeared on the screen of the force meter was recorded as its single-blind measurements.

A visual analog scale (VAS) was used for the evaluation overall patient satisfaction. The assessment was carried out in the Arabic language, which is recorded at insertion (2 weeks) and 3, 6, and 12 months after prosthesis insertion for all participants. All questionnaires were taken by the same research interviewer (assisted interviewer) as he was blind about the type of prosthesis (double-blind) as the research interviewer is from another department.

Numerical data were explored for normality by checking the distribution of data and using tests of normality (Kolmogorov-Smirnov and Shapiro-Wilk tests). All the data showed a normal (parametric) distribution. Data were presented as mean, standard deviation (SD), and 95% confidence interval for the mean (95% CI) values. Repeated measures one-way an ANOVA test was used to compare between the groups as well as to study the changes over time within each group. Bonferroni's post-hoc test was used for pair-wise comparisons. The significance level was set at (*P* < 0.05). Statistical analysis was performed with IBM SPSS Statistics for Windows, Version 23.0: IBM Corp.

## 3. Results

**Figure 8:**
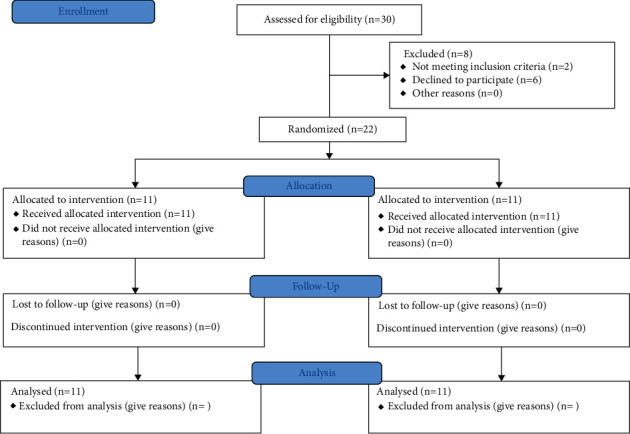
Flow diagram.

The data were collected for all participants during follow-up with no dropouts (Figure 8). There was a decrease in retention throughout the follow-up period in both groups. The PEEK group showed a statistically significantly higher retention force (*P* < 0.05) at the time of insertion (37.6/17.97), 3 months (33.9/16.78), 6 months (32.7/15.72), and 12 months (31.65/13.05) (Table 1). Regarding patient satisfaction, the conventional group showed a statistically significantly (*P* < 0.05) higher mean of overall satisfaction than PEEK (65/82.18) at denture insertion. After 3 months (87.81–84.72), there was no statistically significant difference between both groups. After 6 months (86.36–80.18) and 12 months (85.45–79.54), the conventional group showed a statistically significantly lower mean overall satisfaction than the PEEK group (Table 2).

## 4. Discussion

The use of PEEK housing is clinically promising, which supports the study hypothesis. The introduction of implants in completely edentulous patients have proven their success again and again in providing the denture wearer satisfaction and confidence in their implant-supported overdenture [31].

Previous investigations of attachments are in general agreement that the patients managed with overdentures need regular recalls and continuous maintenance as the loss of retentive force over time is inevitable [32, 33]. This loss of retention has been attributed to wear of attachment components, which may be related to deformation that occurs during insertion, and removal of the prosthesis [34]. Studies on the retentive properties of overdenture attachments concluded that attachments gradually lose their retention. Nylon housings for ball attachments are susceptible to wear, fatigue, and loss of retention [35, 36].

PEEK is a newly introduced material to the field of dentistry; its use as a retentive cap is still widely under investigation. The amount of retention of an overdenture is dependent upon many factors such as arch form, masticatory habits, and type of attachments, where the flexibility of the attachment is a primary determinant of retention. Due to the reduced flexibility of the PEEK as compared to the nylon caps, they showed higher levels of retention, although loss of retention in the PEEK group as compared with the conventional group is higher. This can be logically explained by the increased friction and hence the wear of the caps over time.

Regarding patient satisfaction with overdentures, it depends on multiple factors, like patient preferences, chewing comfort, phonetics, and aesthetics. There is also a direct relationship with the retention of the overdenture [37].

In the current study, a patient satisfaction questionnaire was used as a method of assessment of patient satisfaction as satisfaction measures are associated with oral health-related quality of life, which detects clinically significant differences between various prosthodontic management methods [38]. The participants showed a statistically significant increase in patient satisfaction regarding all aspects. The participants also showed continuous satisfaction throughout the study period, although the retentive properties of the retentive caps of both groups showed a loss in retention. This could be justified by the fact that the patients usually have amazing adaptive qualities, that although the dentures are less retentive, they can still control and adapt to them by musculoskeletal endurances as long as the retentive values for both groups were still within the clinically accepted range. The initial low satisfaction level of the PEEK group may be attributed to the initial high retentive force, which is annoying to patients during the insertion and removal. As the retentive force decreased over time, the patient satisfaction improved within a limit while in the conventional group, decrease of satisfaction may be attributed to the decrease in the retentive force, which negatively effects several aspects.

## 5. Conclusions

Within the limitation of this comparative study, the following conclusions were made:

The PEEK retentive material provided more retention than did the conventional material and led to improved patient satisfaction.

### 5.1. Limitation

The need for a milling machine to mill the cap and the software to handle the files.

### 5.2. Recommendation

Due to the standardization of ball abutment, it is recommended for each company to have its STL file for cap production or have one become available in the market in order to save time and money.

## Figures and Tables

**Table 1:** Comparison between retention forces (*N*) in the two groups.

	PEEK		Ball		*P* value	C.I 95%	Effect size (partial eta squared)
	Mean	SD	Mean	SD			
Zero	37.6^A^	0.992	17.79^A^	0.488	0.001	20.5053–19.1147	0.968
3	33.9^B^	0.917	16.781^B^	0.481	0.001	17.8203–16.5177	0.950
6	32.7^C^	1.015	15.972^C^	0.763	0.001	−17.5266–15.9294	0.523
12	31.65^D^	1.166	13.054^D^	0.643	0.001	−19.4335–17.7585	0.876
Effect size (partial eta squared)	0.979		0.968				

^*∗*^Significant at *P* ≤ 0.05, different superscripts (A, B, C, & D) in the same column are statistically significantly different.

**Table 2:** Comparison between overall satisfaction in the two groups.

	PEEK		Ball		*P* value	C.I 95%	Effect size (partial eta squared)
	Mean	SD	Mean	SD			
Zero	65^A^	5.916	82.18	4.833	0.001	12.3754/21.9846	0.645
3	87.81^B^	3.945	84.72^A^	3.495	0.0660	−7.7824/−0.2016	0.015
6	86.36^B^	5.045	80.18	6.096	0.0175	−11.1567/−1.2033	0.083
12	85.45^B^	5.222	79.54^B^	5.007	0.0135	−10.4601/−1.3599	0.623
Effect size (partial eta squared)	0.821		0.783				

^*∗*^Significant at *P* ≤ 0.05, different superscripts in the same column (A & B) are statistically significantly different.

## Data Availability

The data are available upon request to the corresponding author.

## Additional Points

This study provided support for using PEEK as a housing material to improve implant-supported mandibular overdenture retention as well as patient satisfaction.
